# Allogeneic umbilical cord-derived mesenchymal stem cell transplantation for treating chronic obstructive pulmonary disease: a pilot clinical study

**DOI:** 10.1186/s13287-020-1583-4

**Published:** 2020-02-13

**Authors:** Phuong Le Thi Bich, Ha Nguyen Thi, Hoang Dang Ngo Chau, Tien Phan Van, Quyet Do, Hung Dong Khac, Dong Le Van, Luc Nguyen Huy, Khan Mai Cong, Thang Ta Ba, Trung Do Minh, Ngoc Vu Bich, Nhat Truong Chau, Phuc Van Pham

**Affiliations:** 1 Van Hanh General Hospital, Ho Chi Minh City, Viet Nam; 2 Vietnam Millitay Academy 103, Ha Noi, Viet Nam; 3 Stem Cell Institute, VNUHCM University of Science, Ho Chi Minh City, Viet Nam; 4 Laboratory of Stem Cell Research and Application, VNUHCM University of Science, Ho Chi Minh City, Viet Nam

**Keywords:** Umbilical cord-derived mesenchymal stem cells, Mesenchymal stem cells, COPD, Chronic obstructive pulmonary disease

## Abstract

**Introduction:**

Chronic obstructive pulmonary disease (COPD) is the third leading cause of death worldwide. COPD results from chronic inflammation of the lungs. Current treatments, including physical and chemical therapies, provide limited results. Stem cells, particularly mesenchymal stem cells (MSCs), are used to treat COPD. Here, we evaluated the safety and efficacy of umbilical cord-derived (UC)-MSCs for treating COPD.

**Methods:**

Twenty patients were enrolled, 9 at stage C and 11 at stage D per the Global Initiative for Obstructive Lung Disease (GOLD) classification. Patients were infused with 10^6^ cells/kg of expanded allogeneic UC-MSCs. All patients were followed for 6 months after the first infusion. The treatment end-point included a comprehensive safety evaluation, pulmonary function testing (PFT), and quality-of-life indicators including questionnaires, the 6-min walk test (6MWT), and systemic inflammation assessments. All patients completed the full infusion and 6-month follow-up.

**Results:**

No infusion-related toxicities, deaths, or severe adverse events occurred that were deemed related to UC-MSC administration. The UC-MSC-transplanted patients showed a significantly reduced Modified Medical Research Council score, COPD assessment test, and number of exacerbations. However, the forced expiratory volume in 1 s, C-reactive protein, and 6MWT values were nonsignificantly reduced after treatment (1, 3, and 6 months) compared with those before the treatment.

**Conclusion:**

Systemic UC-MSC administration appears to be safe in patients with moderate-to-severe COPD, can significantly improve their quality of life, and provides a basis for subsequent cell therapy investigations.

**Trial registration:**

ISRCTN, ISRCTN70443938. Registered 06 July 2019

## Introduction

Chronic obstructive pulmonary disease (COPD) was the third leading cause of death in the USA in 2005 (https://www.cdc.gov/copd/basics-about.html). According to the World Health Organization (WHO) estimates, 65 million people worldwide have moderate-to-severe COPD. In 2005, more than 3 million people died of COPD, accounting for 5% of all deaths that year. Numbers of COPD patients are expected to increase by more than 30% in the next 10 years (https://www.cdc.gov/copd/basics-about.html), and COPD is expected to be the third leading cause of death worldwide in 2020. COPD is treated with medications, including bronchodilators, inhaled steroids, oral steroids, phosphodiesterase-4 inhibitors, theophylline, and antibiotics; lung therapies, such as oxygen therapy and pulmonary rehabilitation programs; and surgeries, including lung-volume reduction surgery, lung transplantation, and bullectomy. However, these therapies have limited efficacy and severe adverse effects [1–3]. Stem cell therapy, especially with mesenchymal stem cells (MSCs), is a promising therapy for treating various diseases, including inflammation and autoimmune diseases [4–6].

MSCs are adult stem cells often used to treat diseases such as graft-versus-host disease (GVHD) [5], osteoarthritis [7], autoimmune diseases [8], and liver cirrhosis [9]. Several off-the-shelf mesenchymal stem-cell therapies have been approved as drugs for some diseases. These include Prochymal for GVHD (in Canada) [10], Cartistem for knee osteoarthritis (in South Korea) [11, 12], and Temcell HS for GVHD (in Japan) [13]. MSCs can be derived from different sources such as bone marrow [14–16], adipose tissue [17–20], peripheral blood [21, 22], umbilical cord blood [23, 24], and umbilical cord tissue [25–27]. MSCs have three beneficial therapeutic mechanisms. First, MSCs can modulate the host immune system by inhibiting some immune cells and stimulating others [28–30], thus participating in regulating the immune system. This is the main mechanism of the MSCs used to treat GVHD, autoimmune diseases and inflammatory diseases [31–36]. Recent studies have shown that MSCs can directly interact with immune cells and secrete cytokines or interleukins to regulate host immune cells [37–40]. The second mechanism relates to the MSC secretome. MSCs can produce a wide variety of cell signaling cytokines and *growth factors* targeting endogenous stem cell self-renewal and migration [41–44] and can trigger host stem cells to self-renew and differentiate to heal an injury. Finally, MSCs can home and differentiate after transplantation [45–47]. In some cases, particularly, in autologous transplantation, MSCs can home and reestablish stem cell niches in the host. These MSCs can differentiate into functional cells that participate in tissue regeneration.

Moreover, MSCs are of interest for therapies using adult stem cells because they can be used in allogeneic transplantation cases that are not HLA-matched between stem cells and recipients. MSCs express low levels of human leukocyte (HLA) class I [48, 49]. They also do not express HLA class II or costimulatory molecules, including CD40, CD80, and CD86, which are essential for T cell immune responses [48, 49].

MSCs have been applied in both autologous and allogeneic transplantations in animals and humans to treat diseases, including COPD. The first allogeneic MSC transplantation was the application of prochymal to treat COPD. Prochymal is the first allogeneic off-the-shelf stem cell treatment produced from human bone marrow. This product was approved as a drug in Canada in 2012 to treat GVHD. A report from Osiris Therapeutics showed that prochymal transplantation provided some benefits without adverse effects in 62 COPD patients but did not improve their quality of life or lung function [50]. Other studies have used MSCs derived from bone marrow (BM) or adipose tissue to treat COPD [51–53]; however, most studies showed limited efficacy [51–53]. The failure of these three clinical trials revealed some issues relating to MSC transplantation for COPD. The first issue may involve the use of frozen MSCs. In the first clinical trial (NCT00683722), frozen BM-MSCs were thawed and directly infused into patients immediately after thawing in frozen bags [50]. The off-the-shelf BM-MSCs were produced on an industrial scale as stem-cell drugs. Although this product enables easy and convenient transplantation, a recent report showed that newly thawed MSCs lose part of their immunomodulatory capacity [54]. Similarly, in the second clinical trial (NCT01306513), the newly thawed cells were also directly used to treat patients but with low efficacy [51, 52]. Thus, fresh cultured BM-MSCs should be used instead of newly thawed BM-MSCs. However, a newer clinical trial (NCT01110252) used fresh cultured BM-MSCs but yielded no improvement in clinical outcomes [53]. Thus, autologous BM-MSCs may be unsuitable for treating COPD. BM-MSCs are usually isolated from adult patients, and BM-MSCs from aging patients can function abnormally compared with MSCs derived from younger tissues. In animals, BM-MSCs from aged animals have shorter telomere lengths, reduced differentiation capacity, impaired proliferation, and decreased paracrine factor production compared with those from younger animals [55–57]. In mouse models, BM-MSCs from aged mice showed downregulated cytokine and chemokine receptor expression. These BM-MSCs were also less mobilized to lung injury compared with BM-MSCs derived from younger mice [58]. Human BM-MSCs from aged patients highly express senescence-related genes, shorter telomere length, low proliferation and low differentiation capacity [59]. In summary, BM-MSCs appear unsuitable for COPD treatment.

In contrast to BM-MSCs, umbilical cord-derived MSCs (UC-MSCs) exhibit strong modulation capacity, and under the same conditions, we found that UC-MSCs more strongly inhibited allogeneic lymphocytes than did BM-MSCs or adipose tissue-derived mesenchymal stem cells [60–62]. UC-MSCs also have higher proliferation rates, are more primitive than are BM-MSCs [63, 64], and exhibit better potential to differentiate into other cells [63–66]. Thus, we hypothesize that UC-MSCs are suitable MSC sources for COPD treatment. Therefore, this study evaluated the efficacy and safety of using expanded allogeneic MSCs from human umbilical cord tissue to treat COPD.

## Materials and methods

### Study design and oversight

This was a pilot clinical trial, without a control group. The institutional review board (scientific and ethical committee) of Van Hanh General Hospital (Ho Chi Minh City, Viet Nam, no. 084/2017/QD-NCKH) and Vietnam Military Academy 103 (Hanoi, Vietnam) approved the study, and all participants provided written informed consent. The study was conducted in accordance with the amended Declaration of Helsinki.

### Patient selection

**Fig. 1:**
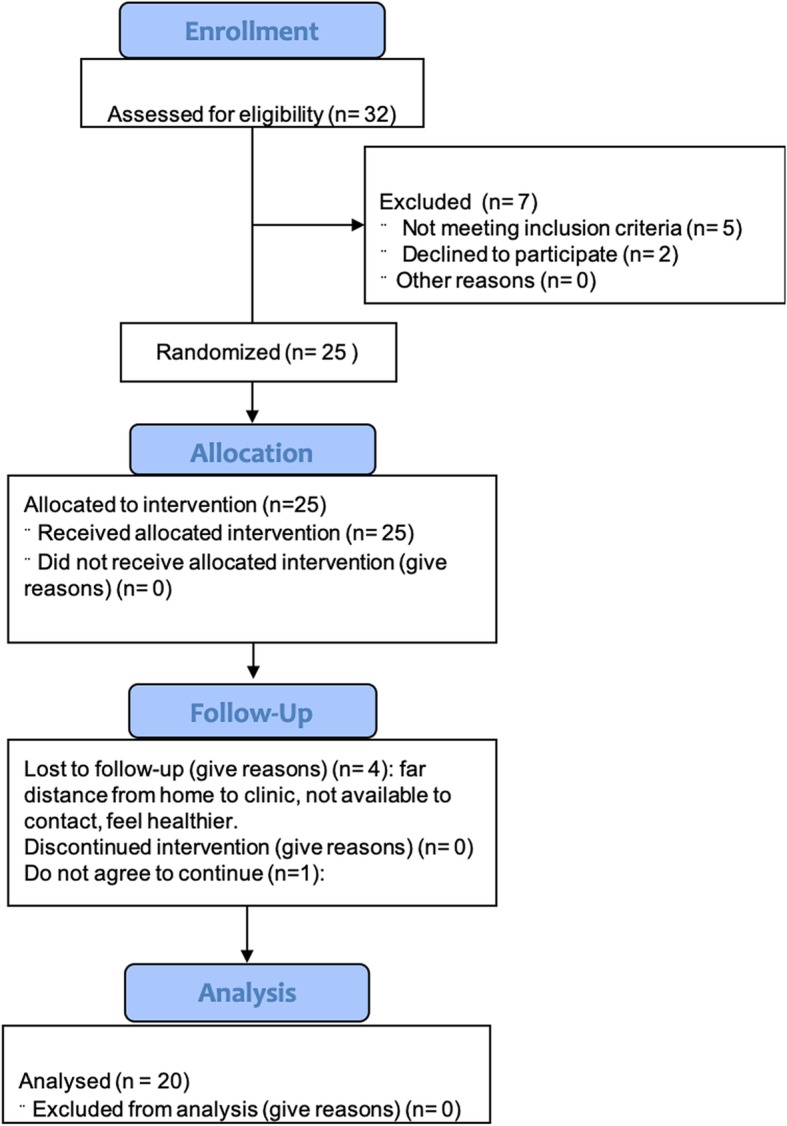
Flow chart of the study population selection including patient recruitment, exclusion criteria, and refusals

Eligible patients were aged 40–80 years with moderate-to-severe COPD at stage C or D per the Global Initiative for Chronic Lung Disease (GOLD), had a smoking history of > 10 pack-years, a postbronchodilator forced expiratory volume in 1 s (FEV_1_)/forced volume capacity (FVC) ratio < 70%, and a postbronchodilator FEV_1_ between 30 and 70% of the predicted value (Fig. 1).

The inclusion criteria were as follows:

1. Diagnosed with COPD stage C or D in accordance with GOLD 2016;
2. Aged 40–80 years;
3. Understood and agreed to the written consent form.

The exclusion criteria were as follows:

1. Current smoker or smoking cessation time of less than 6 months;
2. Asthma or clinically relevant lung disease other than COPD (lung tuberculosis, restrictive lung disease, idiopathic pulmonary fibrosis, or lung cancer);
3. Active infection requiring antibiotic therapy;
4. Active mycobacterial infection;
5. Clinical relevance unassociated with COPD during screening: left ventricle ejection fraction lower than 40%, valvular heart disease, cardiomyopathy, arrhythmia, congenital heart disease, kidney failure with creatinine index > 2.0 mg/dl, liver disease with AST, ALT or bilirubin twice the upper limit of the normal range, hematological disorder, or cancer;
6. Using a tumor necrosis factor inhibitor within 3 months of the screening visit;
7. Using an immunosuppressive medication within 8 weeks of the screening visit;
8. Active malignancy or history of cancer without recurrence within 5 years prior to screening visit;
9. Participating in other clinical trials with any medication or medical device;
10. Being unable to perform all assessments required for the study.

### Isolation of umbilical cord-derived mesenchymal stem cells

The umbilical cord-derived mesenchymal stem cells (UC-MSCs) were cultured and expanded using the UC-SCI technology developed by the Stem Cell Institute, VNUHCM University of Science, Ho Chi Minh City, Viet Nam. This technology permits isolating and expanding UC-MSCs from Wharton’s jelly and whole umbilical cord tissue. The culture procedure is serum-free and xeno-free.

**Fig. 2:**
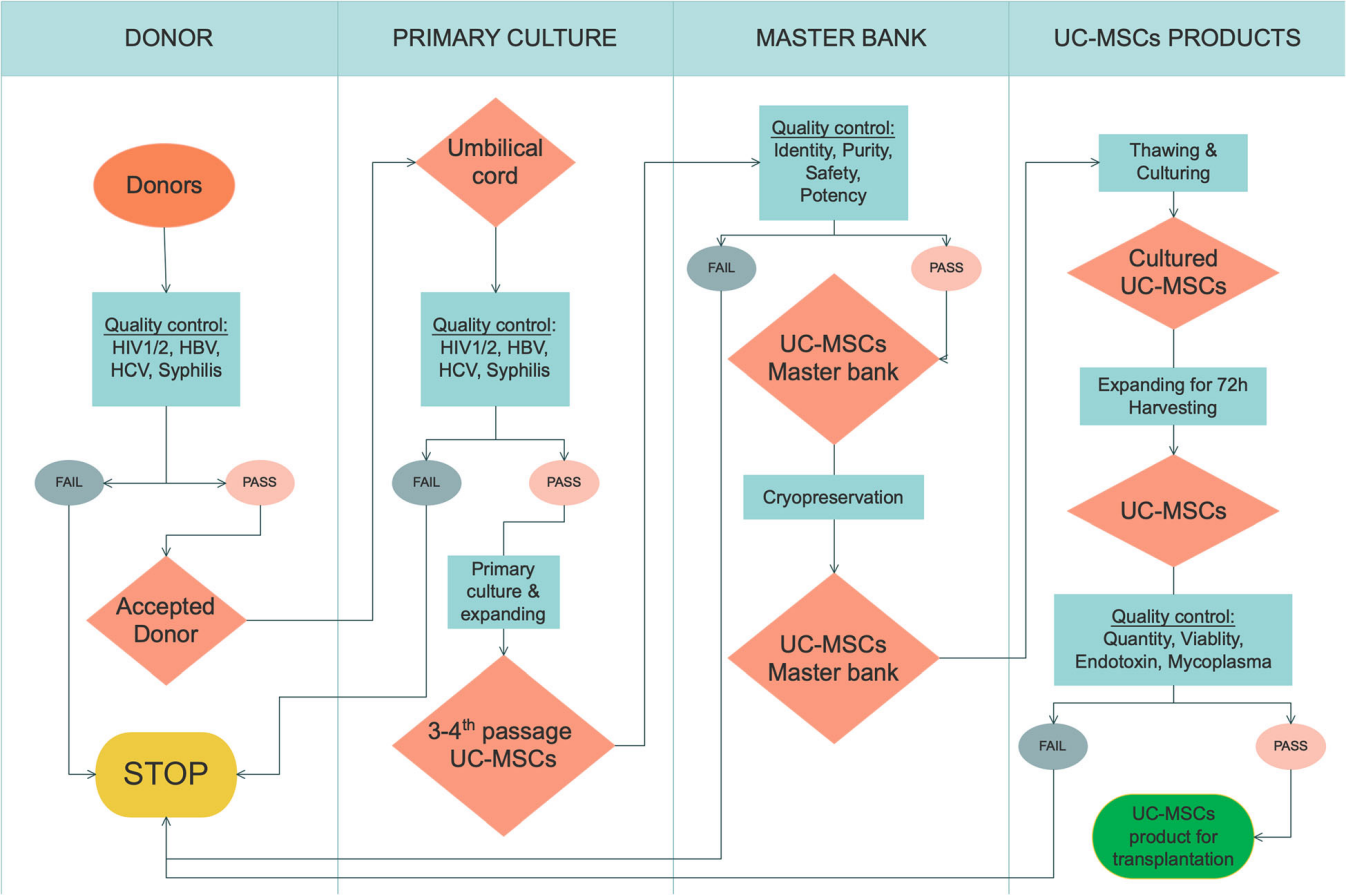
Work-flow of UC-MSC production for clinical application. Donors were screened to select suitable donors for umbilical cord collection. Umbilical cord tissue was used to isolate UC-MSCs by primary culture; them UC-MSCs were expanded before they were freezed. During the process, UC-MSCs were checked to control the UC-MSC quality

Umbilical cord samples were collected from donors during childbirth as approved by the local ethics committee. The criteria for umbilical cord tissues were that the tissues were from a full-term birth and negative for HIV½, HBV, HVC, and syphilis. All samples used to isolate the UC-MSCs met these criteria (Fig. 2).

Umbilical cord samples were cut into 1-cm-long fragments, then the veins and artery inside were removed, washed twice with washing buffer (PBS), then cut into 1–2 mm^3^ fragments. These fragments were placed in a T-75 flask with Wharton’s jelly contacting the flask surface. Finally, 4 mL of the MSCCult I medium (DMEM/F12, supplemented with 7.5% activated platelet-rich plasma derived from the umbilical cord blood according to the published protocol [25]) was added to each T-75 flask and placed in an incubator at 37 °C and 5% CO_2_. After 3 days, 6 mL of fresh MSCCult I medium was added to the T-75 flask. The 100% culture medium was changed with 10 mL of fresh culture medium after 7 days. When the cells migrated to the fragments and reached 70% confluence, the tissues were removed, and the cells were subcultured via detachment reagent (Deattachment Solution, Regenmedlab). The UC-MSCs were continuously expanded through 5 passages to obtain sufficient pure cells for transplantation.

### Quality control of the UC-MSC master bank

After 3–4 passages of the UC-MSCs, 4 flasks were randomly selected from all UC-MSC flasks for quality control. The other flasks were used to collect all UC-MSCs for cryopreservation as the UC-MSC master bank. Quality control for the UC-MSC master bank included identity, purity, safety and potency evaluations.

#### Identity and purity

The UC-MSCs were confirmed as MSCs per ISCT recommendations for MSCs. MSCs were observed under an inverted microscope to evaluate their shape. Their phenotypes were evaluated for CD14, CD34, CD44, CD45, CD73, CD90, CD105, and HLA-DR expressions. The protocols for immunophenotyping followed the published protocol [67]. Briefly, cells were washed twice in PBS containing 1% bovine serum albumin (Sigma-Aldrich, St. Louis, MO, USA). The cells were then stained with anti-CD14-FITC, anti-CD34-FITC, anti-CD44-PE, anti-CD45-FITC, anti-CD73-FITC, anti-CD90-PE, anti-CD105-FITC, or anti-HLA-DR-FITC antibodies (all antibodies were purchased from BD Biosciences, San Jose, CA, USA). Stained cells were analyzed via FACSCalibur flow cytometer (BD Biosciences). Isotype controls were used in all analyses. The purity was calculated based on the percentage of UC-MSCs that were positive for CD44, CD73, CD90, and CD015 and negative for CD14, CD34, CD45, and HLA-DR. The last assay was an in vitro differentiation of the UC-MSCs. This UC-MSC sample was re-evaluated for in vitro differentiation toward adipogenic cells, chondroblasts, and osteoblasts using osteogenic, osteoblast, and chondroblast differentiation kits from Thermo Fisher Company (Thermo-Fisher, Waltham, MA, USA).

#### Safety

UC-MSCs were evaluated for sterility according to USP71, mycoplasma according to USP63, endotoxins according to USP85, and viral infections (HIV 1/2, HBV, HCV) via real-time PCR using commercial kits. These cells also were evaluated for in vivo tumorigenicity. The in vivo tumorigenicity was tested by injecting UC-MSCs under the skin of 3 NOD/SCID mice using GFP-expressing breast cancer stem cells as positive controls (Stem Cell Institute, VNUHCM University of Science). Both breast cancer stem cells and UC-MSCs were injected into the NOD/SCID mice at 5 × 10^6^ cells/100 μl. UC-MSCs were injected on the right side, while breast cancer stem cells were injected into the left abdominal area. Tumor formation was observed macroscopically for 30 days and checked via in vivo imaging to visualize the positive control using the Extreme II system (Bruker). After being injected with the cells, the mice were continuously monitored for tumor development for 3 months before stopping the experiments.

#### Potency

The UC-MSC potency was evaluated by inhibiting the UC-MSCs on allogeneic lymphocyte proliferation as per the published protocol [68]. To prepare the peripheral blood mononuclear cells (PBMCs), PBMCs from the biobank were thawed and incubated in RPMI 1640 medium containing 10% fetal bovine serum (FBS) at 37 °C and 5% CO_2_ for 3 h to recover, then collected and labeled with CellTrace™ CFSE (Thermo-Fisher, Waltham, MA, USA) per the manufacturer’s instructions. The UC-MSCs were prepared by seeding them at 3000 cells/cm^2^ in 6-well plates in the MSCCult I medium, then allowed to reach 80% confluence. The labeled PBMCs were added to MSCs in 6-well plates at a 1:10 ratio (MSC:PBMCs). Next, phytohemagglutinin was added to the RPMI in the wells (PBMCs + MSCs) to a final concentration of 20 μg/mL per well. The negative control for this assay was 250,000 PBMCs per well in a six-well plate with complete RPMI medium; the positive control was 250,000 PBMCs per well in a 6-well plate with complete RPMI medium supplemented with a final concentration of 20 μg/mL phytohemagglutinin. After 3 and 5 days, 3 wells from the 6-well plates were collected to analyze the proliferating T cell population. The T-cell proliferation percentage was calculated based on the negative or lower CFSE signal compared with the positive and negative control samples. All samples were analyzed via flow cytometer (FACSCalibur; BD Bioscience, Franklin Lakes, NJ, USA) with CellQuest Pro software.

### Preparation of UC-MSCs for transplantation

The thawed UC-MSCs were cultured for 72 h, then harvested for transplantation, isolated with deattachment reagent, washed twice with washing buffer, and resuspended in saline solution for transfusion. The quantity and viability of the UC-MSCs were evaluated using a C6 Accuri flow cytometer (BD Bioscience). Mycoplasma contamination was evaluated according to USP63, and endotoxins according to USP85. Cell viability was evaluated based on 7-AAD expression and detected by the FL3 channel in the flow cytometer (BD Accuri C6, BD Bioscience). Data were collected and analyzed using CellQuest Pro software.

The population doubling time (DT) of UC-MSCs after thawing and recovery was determined. The doubling time was calculated using the formula DT = (t2-t1)ln(2)/ln (n2/n1) where n2 is the cell number at harvesting, n1 is the cell number at plating, t2 is the time at cell harvest and t1 is the time at plating.

### Study treatments and outcome evaluations

Enrolled patients were intravenously infused with 1.5 × 10^6^ fresh allogeneic MSCs/kg directly harvested from the T-75 flasks on day 0. UC-MSCs were delivered at a maximum rate of 2.0 × 10^6^ cells/min. Each infusion took approximately 45 min to complete.

Participants were subsequently evaluated for safety and efficacy at 1, 3, and 6 months. Safety was assessed by the occurrence of adverse events during either the study or the drug infusion by physician assessments, laboratory evaluations, and electrocardiograms (ECGs), as well as ECGs during the 6-month follow-up period.

A record of COPD exacerbations was maintained for each patient. Efficacy measures included improvement from baseline in pulmonary functions (FEV_1_, FVC, FEV_1_/FVC, total lung capacity by plethysmography, single-breath carbon monoxide diffusing capacity, exercise performance (6-min walk test [6MWT]), dyspnea assessment (Borg scale), and quality of life (St. George’s Respiratory Questionnaire and global assessment of patient status). COPD exacerbations were assessed as the time to first exacerbation and the ratio of the rate of exacerbations in UC-MSC-treated patients.

### Statistical analysis

The number of patients was selected for the initial safety assessments and exploratory evaluation of efficacy. For all other end-points, statistical analyses were performed using two-sided hypothesis tests, including *t* tests, *χ*^*2*^ tests, Wilcoxon rank-sum tests, or Fisher’s exact tests as appropriate, at the 0.05 significance level. Differences in time to first COPD exacerbation and probabilities of being exacerbation-free were assessed via Kaplan-Meier methodology and log-rank tests. Total COPD exacerbations experienced per patient and adjusted per exposure were compared between treatment groups using a two-sided Mantel-Haenszel *χ*^*2*^ test for ordered categorical data.

## Results

### Isolation of UC-MSCs and establishment of UC-MSC master bank

After the 4th subculture, UC-MSCs were used to perform control quality for the master bank with identity, purity, safety, and potency assays.

**Fig. 3:**
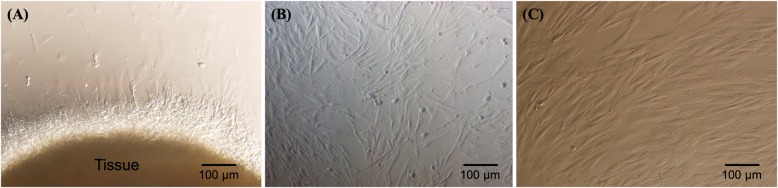
UC-MSC culture and expansion. The MSCs migrated from the tissue after 7 days of primary culture (**a**). They were subcultured for UC-MSC mater banks (**b**). The UC-MSCs were thawed and cultured for transplantation (**c**)

**Fig. 4:**
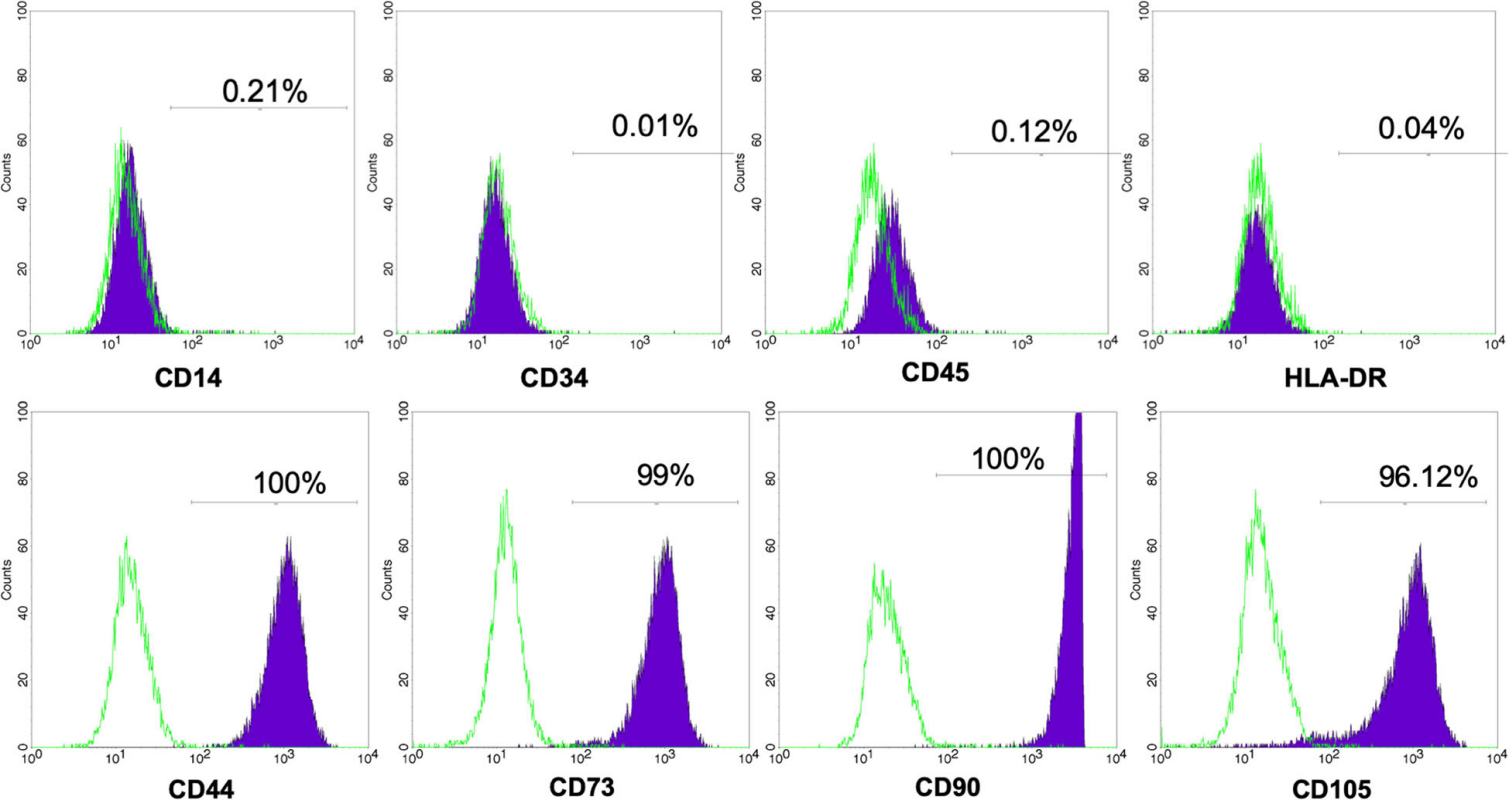
UC-MSCs expressed the common markers of MSCs suggested by ISCT. They expressed CD44, CD73, CD90, and CD105; and did not express CD14, CD34, CD45, and HLA-DR

UC-MSCs adhered well to the plastic flask surface and exhibited a fibroblast-like shape (Fig. 3). At passage 4, the cells showed expression of the common MSC markers, CD44 (100%), CD73 (98 ± 0.11%), CD90 (100%), and CD105 (95.42 ± 2.13%). However, the UC-MSCs were negative (or low) for the hematopoietic markers, CD14 (0.21 ± 0.04%), CD34 (0.013 ± 0.09%), CD45 (0.12 ± 0.10%), and HLA-DR (0.04 ± 0.01%) (Fig. 4).

**Fig. 5:**
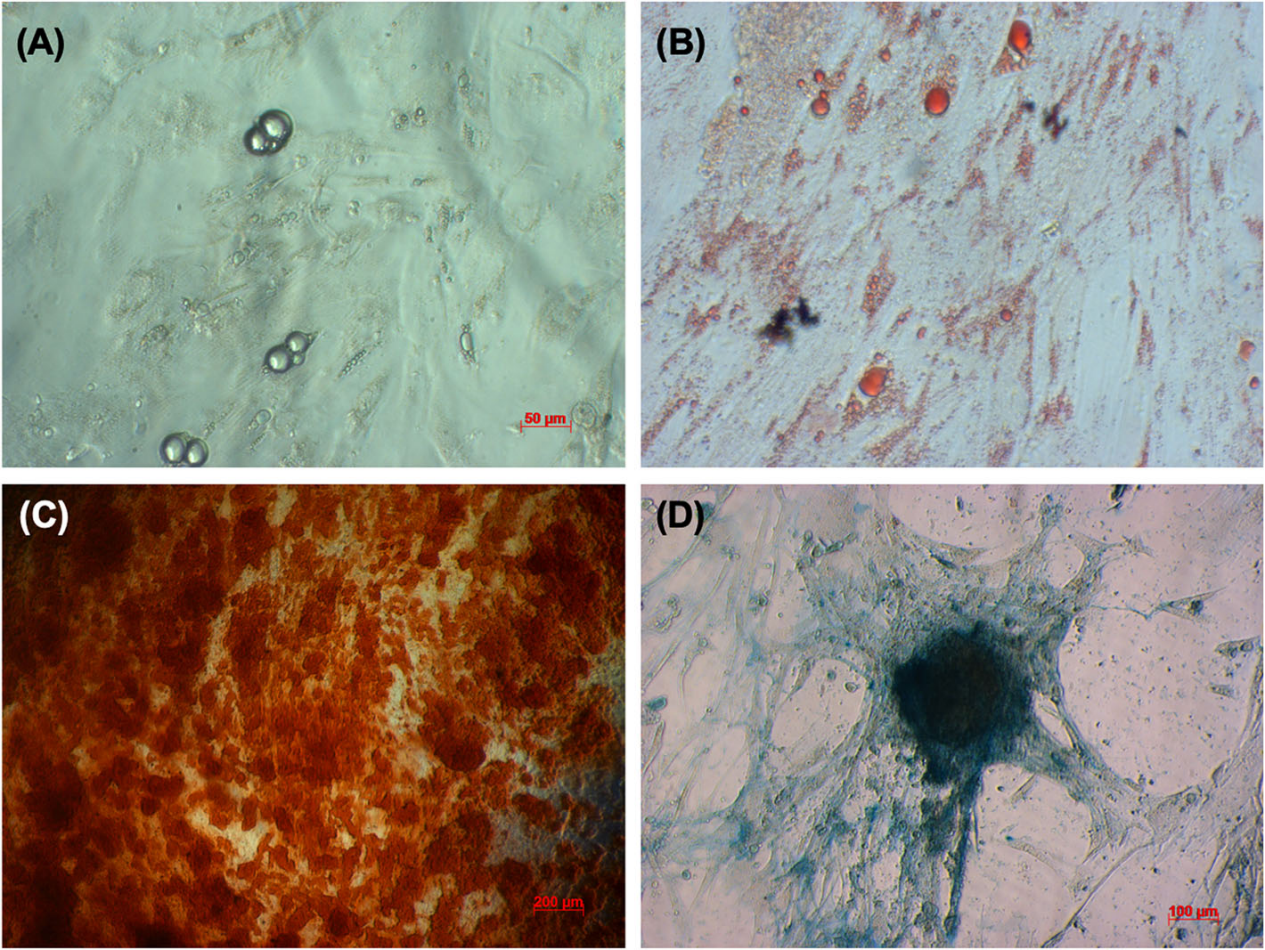
UC-MSCs were successfully differentiated into adipocytes, osteoblasts, and chondroblasts. After differentiation, UC-MSCs successfully differentiated into adipocytes (**a**) that were positive with Oil Red staining (**b**); into osteoblasts that positive with Alizarin red staining (**c**); into chondroblasts that positive with Alcian blue staining (**d**)

UC-MSCs were successfully differentiated into osteoblasts, adipocytes, and chondroblasts. In the induced medium geared toward osteoblast differentiation, UC-MSCs gradually changed their morphology to a longer shape and gradually produced more matrix. After 21 days of induction, the differentiated samples stained positive with Alizarin red. The dyes combined with calcium in the matrix and displayed the red color. UC-MSCs were also successfully differentiated into adipocytes. These cells accumulated lipid droplets in the cytoplasm, which were stained with oil red O. Differentiation of UC-MSCs into chondroblasts was also recorded in vitro after inducing MSCs for 21 days in the induced medium. Overexpression and accumulation of proteoglycans and collagen I were evaluated in these differentiated cells by alcian blue staining (Fig. 5).

The mycoplasma assay showed that all UC-MSC samples were negative for mycoplasma; endotoxins were < 0.025 EU/mL. The culture supernatant was negative for HIV1/2, HBV, and HCV. After 30 days and 3 months, the UC-MSCs caused no tumors in the NOD/SCID mice.

**Fig. 6:**
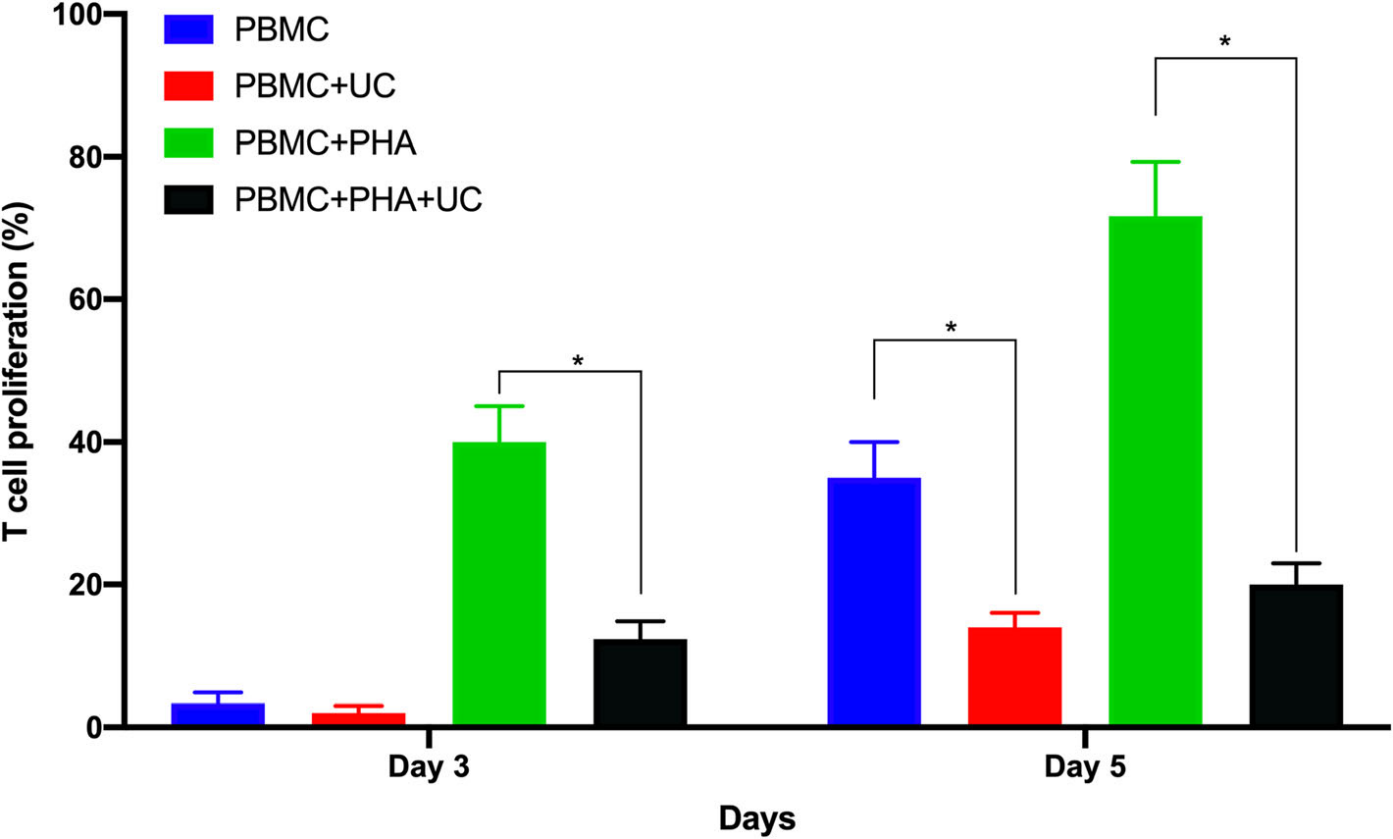
UC-MSCs can inhibit T cell proliferation after 3 and 5 days co-culture. After 3 days of co-culture with PBMC, UC-MSCs can efficiently PHA-treated PBMC proliferation. After 5 days of co-culture, UC-MSCs can strongly inhibit the both PHA-treated PBMC and non-treated PBMC proliferation

Figure 6 shows the UC-MSC potency, indicating that the UC-MSCs efficiently inhibited T cell proliferation. Proliferation of PHA-treated T cells was significantly reduced when cocultured with UC-MSCs for 3 and 5 days. On day 3, T cell proliferation percentages were 3.33 ± 1.53%, 2.00 ± 1.00%, 40.00 ± 5.00%, and 12.33 ± 2.52%; respectively, for PBMC, PBMC+UC, PBMC+PHA, and PBMC+PHA + UC groups. UC-MSCs could efficiently inhibit T cell proliferation (*p* < 0.05) (12.33 ± 2.52% in PBMC+PHA + UC group vs 40.00 ± 5.00% in PBMC+PHA group). After 5 days, T cell proliferation percentage significantly increased in PBMC group compared to PBMC+UC group (35 ± 5.00% vs. 14.00 ± 2.00%, respectively); and in PBMC+PHA compared to PBMC+PHA + UC group (*p* < 0.05) (71.67 ± 7.64% vs. 20.00 ± 3.00%). These results showed that UC-MSCs could inhibit T cell proliferation in both PHA and without PHA treatment.

These results showed that the UC-MSC samples satisfied all the criteria for establishing the master bank.

### Preparation of UC-MSCs for transplantation

The UC-MSCs from the master bank were thawed and recovered for 72 h. The doubling time was determined to control cell recovery. The DT of UC-MSCs after 72 h culture was 40.56 ± 1.68 h, similar to before cryopreservation (*p* > 0.05). All samples were free mycoplasma; and endotoxins were < 0.025 EU/mL. The percentage of viable cells was > 99% for all samples.

### Characterization of patients

Table 1 shows patient characteristics. Twenty men were enrolled in this study; 9 patients were at stage C, and 11 were at stage D. The average age of the patients was 67 years, with no significant differences between groups for stage C and stage D patients. Most patients (19/20) were smokers with an average of 17.5 pack-years. Disease duration differed significantly between stage C (3.0 years) and stage D (11 years) patients.

**Table 1:** Baseline characteristics of the study patients

	All patients (*n* = 20)	Stage C (*n* = 9)	Stage D (*n* = 11)
Male sex, *n* (%)	20 (100%)	9 (100%)	11 (100%)
Age, years	67 (55, 81)	65 (59, 81)	68 (55, 81)
Disease duration, years	6.0 (1, 25)	3.0 (1, 10)	11 (2, 25)
Smoker/former smoker, *n* (%)	19 (95%)	8 (88.9%)	11 (100%)
Smoking amount, pack-years*	17.5 (0.0, 70.0)	15.0 (0.0, 65.0)	20.0 (3.0, 70.0)
Had quit smoking, *n* (%)*	19 (100%)	8 (100%)	11 (100%)
Time since quitting smoking, years*	9.5 (0.0, 39.0)	6.0 (0.0, 39.0)	10.0 (3.0, 24.0)

### Safety outcomes

UC-MSC infusions were well tolerated, and no serious or clinically significant adverse events were observed over the course of the study or drug infusions for all patients. No significant changes in oxygen saturation or heart rate were observed during the infusions (Table 2).

**Table 2:** Incidence of adverse events

System organ class/preferred term	Subjects, no.	
	Stage C (*n* = 9)	Stage D (*n* = 11)
Cardiac disorders	0	0
Congestive heart failure	0	0
Gastrointestinal disorders	0	0
GERD	0	0
Peripheral edema	0	0
Immune system disorders	0	0
Seasonal allergies	0	0
Infections and infestations	0	0
Bronchitis	0	0
Nasopharyngitis	0	0
Pneumonia	0	0
Skin infection	0	0
Upper respiratory tract infection	0	0
Urinary tract infection	0	0
Blood calcium increase	0	0
Transient C-reactive protein increase	3	2
Metabolic and nutritional disorders	0	0
Hyperglycemia	0	0
Type 2 diabetes mellitus	0	0
Transient hypertension	5	4

### Clinical outcomes before treatment and during follow-up according to GOLD stage

Table 3 shows the clinical outcomes. FEV1 was slightly altered before (34%) and after treatment at 1 (35%), 3 (33.0%) and 6 (33.5%) months (*p* > 0.05). No statistically significant differences in CRP or 6MWT were observed for 6 months before and after treatment. The mean CRP level decreased from 3.3 mg/dL before treatment to 2.2, 2.4, and 2.3 (mg/dL) after treatment at 1, 3, and 6 months, respectively. The 6MWT increased from 360.0 in patients before treatment to 380, 360.0, and 380.0 after 1, 3, and 6 months of treatments, respectively (*p* > 0.05).

**Table 3:** Comparison of clinical outcomes before and after treatment

Outcome	Before treatment (*N* = 20)	After 1 month (*N* = 20)	*p* value*	After 3 months (*N* = 20)	*p* value*	After 6 months (*N* = 20)	*p* value*
FEV1 (%)	34.0 (24.6, 49.0)	35.0 (25.5, 55.2)	0.107	33.0 (26.5, 51.0)	0.251	33.5 (27.5, 43.0)	0.239
CRP (mg/dL)	3.3 (1.4, 5.8)	2.2 (1.4, 4.0)	0.444	2.4 (1.2, 6.1)	0.702	2.3 (1.3, 5.3)	0.284
mMRC	1.0 (0.0, 2.0)	0 (0.0, 1.0)	**0.033**	0 (0.0, 1.0)	**0.005**	0 (0.0, 1.0)	**0.017**
CAT	10.5 (5.8, 14.5)	6.5 (2.8, 8.8)	**0.002**	4.0 (2.8, 7.2)	**0.001**	2.0 (1.0, 7.5)	**0.003**
6MWT	360.0 (330.0, 420.0)	380.0 (350.0, 400.0)	0.246	360.0 (347.5, 405.0)	0.521	380.0 (355.0, 420.0)	0.250
Number of exacerbations	2 (2, 4)	–	–	–	–	0 (0, 1)	**< 0.001**

Note: Summary statistic is median (interquartile range). *p value was calculated based on Wilcoxon signed-rank test comparing outcomes before treatment with those at each follow-up (1, 3, and 6 months). *Abbreviations*: *FEV1* forced expiratory volume in 1 s, *CRP* C-reactive protein, *mMRC* Modified Medical Research Council, *CAT* COPD assessment test, *6MWT* 6-min walk test

The mMRC, CAT score, and number of exacerbations decreased significantly after 1, 3, and 6 months compared with those before treatment; this reduction was maintained for 1–6 months after treatment. The mMRC value strongly decreased from 1.0 before treatment to 0.0 after treatment at 1, 3, and 6 months (*p* < 0.05). Similarly, the CAT scores were also significantly reduced from 10.05 before treatment to 6.5 at 1 month after treatment, 4.0 at 3 months after treatment, and 2.0 at 6 months after treatment (*p* < 0.05). The COPD exacerbations were dramatically reduced from 2 before treatment to 0 at 6 months after treatment (*p* < 0.05).

### Efficacy in stage C and D patients

**Fig. 7:**
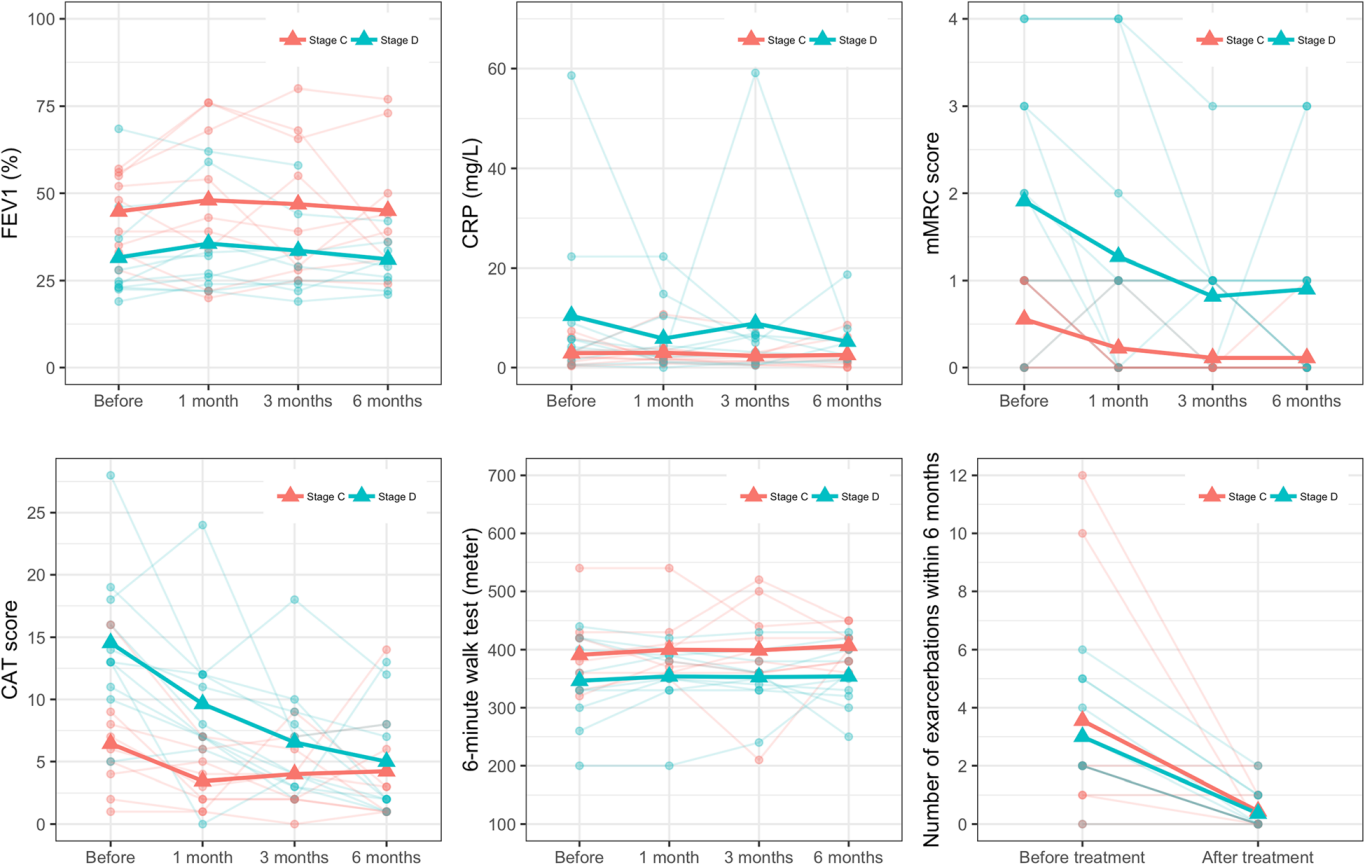
Clinical outcomes before treatment and during follow-up period by GOLD stages (C and D stages)

To evaluate the effects of transplantation on treatment efficacy at stages C and D of COPD, we separated patients into 2 groups, with 9 patients at stage C and 11 at stage D. Figure 7 and Table 4 present the results, which suggest that UC-MSC transplantation yielded better results for stage D COPD patients than for stage C COPD patients.

**Table 4:** Comparison of clinical outcomes before and after treatment in stage C and D patients

Outcome	Before treatment (*N* = 20)	After 1 month (*N* = 20)	*p* value*	After 3 months (*N* = 20)	*p* value*	After 6 months (*N* = 20)	*p* value*
FEV1 (%)							
Stage C	48 (35–55)	43 (34–68)	0.477	39 (29–65.6)	0.516	39 (31–50)	0.922
Stage D	24.8 (23–37)	32 (24–48)	0.079	31 (24–44)	0.309	30 (25–36)	**0.022**
CRP (mg/dL)							
Stage C	2.2 (1.2–3.3)	1.9 (1.5–3.5)	0.914	1.9 (1.2–2.4)	0.547	1.5 (0.8–6.2)	0.688
Stage D	4.2 (1.5–9)	2.9 (2.2–10.4)	0.375	5 (1.1–6.6)	0.966	2.8 (2.3–5.3)	0.426
mMRC							
Stage C	1 (0–1)	0 (0–0)	0.375	0 (0–0)	0.125	0 (0–0)	0.219
Stage D	2 (1–3)	1 (0–2)	0.156	1 (0–1)	**0.031**	0.5 (0–1)	0.094
CAT							
Stage C	6 (4–8)	3 (2–5)	**0.023**	4 (2–6)	0.082	3 (1–6)	0.375
Stage D	13 (11–18)	8 (7–12)	**0.023**	4 (3–9)	**0.001**	2 (2–8)	**0.004**
6MWT							
Stage C	380 (350–420)	380 (360–410)	0.375	400 (360–440)	0.793	400 (380–420)	0.527
Stage D	350 (300–420)	365 (330–390)	0.539	360 (330–380)	0.578	355 (320–400)	0.509
Number of exacerbations							
Stage C	2 (1–2)	–	–	–		0 (0–1)	**0.031**
Stage D	2 (2–5)	–	–	–		0 (0–1)	**0.002**

Stage D COPD patients presented significantly improved mMRC and CAT values after 3 months of treatment and significantly improved FEV1, CAT score, and numbers of exacerbations after 6 months of treatment (*p* < 0.05). The other values did not significantly change after 3 and 6 months (*p* > 0.05; Table 4). After 6 months, the CRP values were reduced by approximately 40% compared with those before treatment in both groups, but this reduction was not significant.

## Discussion

COPD is a chronic inflammatory condition in the lungs, possibly related to smoking. Current treatment for COPD involves the use of anti-inflammatory agents combined with other therapies. However, current therapies have limited efficacy. This study showed that allogeneic non-HLA-matched UC-MSC transplantation is a safe treatment that improved the quality of life of COPD patients. This clinical study was the first to use allogeneic MSCs from umbilical cord tissue to treat COPD.

First, UC-MSCs were isolated and expanded from human umbilical cord tissues. The umbilical cord tissue was carefully checked for viral infections, including HIV1/2, HBV, HCV, and syphilis. Only samples that were negative for these viruses were used to isolate the UC-MSCs. Before the UC-MSCs were used to treat patients, all samples were inspected for quality. The UC-MSCs satisfied all essential criteria for MSCs and cellular products for clinical applications. The MSCs displayed the standard MSC phenotypes: positive for CD44, CD73, CD90, and CD105 and negative for CD14, CD34, CD45, and HLA-DR. They also maintained the capacity to differentiate into osteoblasts, chondrocytes, and adipocytes in vitro. Furthermore, the MSCs were negative for mycoplasma, bacteria, and fungi and were low for endotoxins. The MSC karyotyping was normal (data not shown). The MSC potency was reported previously [60]. The UC-MSCs collected via our protocols exhibited stronger immunomodulation than that of adipose-derived and bone marrow-derived MSCs.

These cells were then infused into COPD patients at 1.5 × 10^6^ UC-MSCs/kg. For 6 months from day 0, all patients at both stages tolerated the MSCs well with no severe or significant adverse effects.

More importantly, the UC-MSC transplantation significantly improved some important outcomes of COPD, including mMCR, CAT, and number of exacerbations. At 1, 3, and 6 months post-transplantation, the CAT and mMCR were significantly reduced (*p* < 0.05), and the number of exacerbations was significantly reduced toward 0 over the 6 months post-transplantation. These clinical outcomes were likely due to downregulated inflammation. The mean CRP decreased from 3.3 mg/dL to 2.3 mg/dL after 6 months; however, this change was not statistically significant, possibly because of the small number of patients in the study. Similarly, the 6MWT score also increased from 360 to 380 but not significantly.

These clinical data showed that UC-MSC transplantation positively affected COPD treatment. In a previous report, we reported two cases of stage D COPD patients who were successfully treated via allogeneic UC-MSC transplantation [69].

To date, this is the first clinical trial to use UC-MSCs in COPD treatment. Several clinical trials registered in clinicaltrials.gov were performed to treat this disease using BM-MSCs or adipose-derived stem cells. Only three of these were completed and reported the results. One trial used allogeneic BM-MSCs, and the other two used autologous BM-MSCs. These clinical trials were performed in moderate-to-severe COPD patients.

The first clinical trial was completed in the USA (NCT00683722) [50]. Sixty-two patients with moderate-to-severe COPD were randomized to intravenously receive an infusion of either ex vivo cultured allogeneic BM-MSCs (Prochymal, Osiris Therapeutics, Inc.) at 100 million MSCs/infusion or a control vehicle. The results showed that during the 2 years of follow-up, the BM-MSC-treated patients had no serious adverse effects or increased COPD exacerbation frequencies. However, although the CRP values decreased in the MSC-treated group, the pulmonary function testing as well as quality of life indicators was not significantly improved compared with those of the nontreatment group [50].

The other two clinical trials (NCT01110252 and NCT01306513) used autologous BM-MSCs to treat COPD patients. In study NCT01110252, 4 patients were transplanted with autologous BM-MSCs and followed for 2 years; no patients experienced adverse effects. Laboratory parameters, clinical conditions, and quality of life were slightly improved [51, 52]. Similarly, in the third clinical trial (NCT01306513), 7 patients were intravenously infused with autologous BM-MSCs [53]. After 1 year of follow-up, no adverse effects related to BM-MSCs were detected, and only one 3-fold increase in CD31 in the alveolar septa was recorded [53].

In contrast to these clinical trials, UC-MSC transplantation significantly improved the quality of life and clinical conditions of COPD patients, possibly due to the strong immunomodulation capacity of the UC-MSCs compared with that of BM-MSCs reported in some publications [60, 70]. This may be due to the anti-inflammatory effects of UC-MSCs, which are stronger immunomodulatory cells than are MSCs from adipose tissue or bone marrow [60]. These cells effectively inhibited the T cells, B cells, and NK cells via various mechanisms [60, 71–73].

UC-MSCs have been reported as promising MSC sources for treating various diseases in humans, including heart failure [74], type 2 diabetes mellitus [75], ankylosing spondylitis [76], and angioplasty for diabetic feet [77].

In this study, we analyzed the efficacy of UC-MSC transplantation in stage C and D COPD patients. Stage D patients responded more strongly to the treatment than did stage C patients. Most clinical outcomes of CODP remained reduced after 6 months of treatment, including the CRP, mMRC score, CAT score, and number of exacerbations, while the 6MWT score was slightly increased in stage D COPD patients.

## Conclusion

In summary, systemic administration of UC-MSCs appears safe. The initial results also showed that UC-MSCs transplantation improved mMRC, CAT scores, and number of exacerbations in an older, comorbid population of moderate-to-severe COPD patients with compromised lung function. Treatment efficacy did not significantly differ between stage C and stage D COPD patients; however, stage D COPD patients exhibited a stronger medical response after UC-MSC transplantation than did stage C COPD patients. Although this is a pilot study, these primary results provide an important and significant basis for further clinical investigations of MSCs in patients with COPD.

## Data Availability

Data and materials used and/or analyzed during the current study are available from the corresponding author on reasonable request.

## Abbreviations

6MWT: 6-min walk test
CAT: COPD assessment test
CRP: C-reactive protein
ECG: Electrocardiogram
FEV1: Forced expiratory volume in 1 s
GVHD: Graft versus host disease
HBV: Hepatitis B virus
HCV: Hepatitis C virus
HIV1/2: Human immunodeficiency viruses 1/2
HLA: Hyman leukocyte antigen
mMRC: Modified Medical Research Council
MSC: Mesenchymal stem cell
UC: Umbilical cord
VNUHCM: Viet Nam National University, Ho Chi Minh city
